# Predicting Quality of Life among Mothers in an Online Health Community for Children with Type 1 Diabetes

**DOI:** 10.3390/children7110235

**Published:** 2020-11-18

**Authors:** Ju-Yeon Uhm, Myoung Soo Kim

**Affiliations:** Department of Nursing, Pukyong National University, Busan 48513, Korea; kanosa@pknu.ac.kr

**Keywords:** type 1 diabetes, children, mothers, quality of life, online health community

## Abstract

Quality of life of parents of children with chronic disease is important for disease self-management. This study aimed to identify predictors of quality of life among mothers of children with type 1 diabetes. A cross-sectional study was conducted. A total of 208 mothers of children with type 1 diabetes were recruited from an online health community. Online health community collective empowerment and social support, diabetes self-efficacy, diabetes-related burden, and quality of life were measured. A multiple regression analysis was conducted to determine predictive factors for quality of life. Multiple regression analysis showed that diabetes-related burden and the child’s age were predictors of quality of life, and total variance explained by the model was 64.1% using two factors. In mothers of younger children, it is important to reduce the diabetes-related burden. Factors that increase the diabetes-related burden should be identified, and strategies to reduce the diabetes-related burden must be established.

## 1. Introduction

Type 1 diabetes (T1D) is a serious chronic childhood disease that requires long-term management. Effective self-management behavior is essential in improving the quality of life (QOL) of children with T1D. Moreover, parents are crucial social determinants of children’s health care [1]. The emotional, behavioral, and cognitive coping strategies of parents are therefore necessary for successful self-management of children with T1D [2,3]. However, up to 74% of parents of children with T1D reported experiencing psychological distress, and 33.5% experienced diabetes-related stress [4]. Additionally, parents reported concerns regarding the child’s hypoglycemia and administering insulin [5], and their worries regarding hypoglycemia were associated with anxiety and negative emotions [6]. Parents specifically experienced greater distress during the early diagnosis stage [7]. Furthermore, parental diabetes-related distress and negative emotions were related to low parental QOL. Parents of two-thirds of pediatric T1D patients reported a low QOL, which was subsequently associated with children’s elevated glycated hemoglobin (HbA1C) levels [8].

Additionally, QOL in the domains of perceived health, vitality, social functioning, and emotional and psychological health among mothers of children with T1D was lower than that of mothers of children without T1D [9]. A further study indicated that all domains of mothers’ QOL were lower, excluding the bodily pain domain [10]. Compared with fathers, mothers’ QOL scores were lower at diagnosis and at one year after diagnosis [11].

Diabetes-related burden [12], emotional support, and children’s dependency on parents were predictors of QOL among mothers of children with T1D [13]. However, parental diabetes self-efficacy was positively correlated with children’s HbA1C and blood glucose monitoring levels [7]. Self-efficacy was a predictor of parental QOL in children with chronic conditions [14]. Furthermore, an increase in mothers’ self-efficacy was associated with higher well-being [15].

Parents of children with T1D struggle to live normal lives [16] and require social support [17]. Parental social support has been shown to positively affect parental self-efficacy [18]. With the increasing use of the internet and smartphones, a growing number of patients and their families have become active in online health communities or online self-help groups. In self-help groups, parents can share their difficulties with others, receive support, and get advice regarding the disease management of their children [19]. Online social support platforms were associated with increased self-efficacy in parents of children with T1D [20]. 

Furthermore, client-centric care emphasizes patient empowerment to improve patients’ health status, experiences, actions, behaviors, and decision-making [21]. Empowerment includes both individual and collective aspects [22]. Collective empowerment is defined as the resolution of health-related issues achieved by working collaboratively with others, or when a group has an effect on the broader societal structure [22,23]. Self-help groups for parents of children with health issues can enhance empowerment and provide a practical strategy to increase self-efficacy [24].

A previous study that included mothers of children with T1D revealed that social support, relationship satisfaction, and general self-efficacy were predictors of QOL and mental health [9]. However, no research exists regarding the association between social support and collective empowerment from the online health community, diabetes self-efficacy, and diabetes-related burden, which are predictors of QOL among mothers of children with T1D. Based on prior literature, a conceptual framework could be established, positing that lower diabetes-related burden [12], higher social support [9,20], and higher self-efficacy [9] may be predictors of higher QOL among parents of children with T1D. Collective empowerment may also be a predictor of higher QOL derived from collaborative aspects of online health communities, and may improve self-efficacy based on descriptive studies that include parents of children with T1D [19,20] or other chronic conditions [24]. As parents play a vital role in promoting children’s health, efforts to improve parental QOL are needed, and interventions to improve QOL should be developed. Therefore, it is necessary to understand the predictors of mothers’ QOL. Thus, this study aimed to investigate predictors of QOL in mothers of children with T1D. We hypothesized that collective empowerment, social support, self-efficacy, and diabetes-related burden would influence the QOL of mothers belonging to an online self-help community.

## 2. Methods

### 2.1. Study Design and Participants

This study utilized a cross-sectional design. Inclusion criteria were as follows: (1) mothers of children with T1D; (2) mothers actively participating in an online T1D health community for longer than six months, and more than six months after the diagnosis of their child; and (3) children in the age range of 6 to 18 years (grades 1 to 12). A screening question was created to identify whether participants met the inclusion criteria before participating in the survey.

To calculate the appropriate effect size, previous studies regarding QOL in mothers of children with T1D were reviewed. Effect sizes were calculated using squared multiple correlation. A ρ^2^ value of 0.36 was obtained in a study including social support and self-efficacy as predictors [9], and 0.27 in a study including school/daily life and fear of hypoglycemia [5] as predictors. Based on the two previous studies, the required sample size was calculated to be 191 to be able to detect an effect size of 0.15, an alpha level of 0.05, and a power of 0.9 using G-power 3.1.9.4.

### 2.2. Measures

#### 2.2.1. Online Health Community Collective Empowerment (OHCE)

The Collective Empowerment in Online Health Communities Scale was used to measure OHCE [25]. The scale comprises two subscales: knowledge for resources (1 to 6 items) and resource mobilization for group action (7 to 11 items). Responses were recorded on a 5-point Likert scale where 1 = “strongly disagree” and 5 = “strongly agree”. The Cronbach’s alpha was 0.94 in Antanasova and Petric’s study (2019), and 0.84 in this study. 

#### 2.2.2. Online Health Community Social Support (OHSS)

A modified Multidimensional Scale of Perceived Social Support (MDSSS) was used to measure OHSS [26]. The scale comprises five items corresponding to the social support of the online health community adapted from the MDSSS [27]. Responses were recorded on a 7-point Likert scale, where 1 indicated poor social support and 7 indicated strong perceived social support. The Cronbach’s alpha was 0.94 in Nambisan’s study (2011) and 0.88 in this study.

#### 2.2.3. Diabetes-Related Burden (DRB) 

The Problem Areas in Diabetes-Parent Revised Scale was used to measure the DRB of parents [12]. This scale comprised 18 items rated on a 5-point Likert scale. Two subscales included immediate burden and theoretical burden. The higher the score, the greater the DRB. The Cronbach’s alpha was 0.87 in the study by Markowitz et al. (2012) and 0.93 in this study.

#### 2.2.4. Diabetes Self-Efficacy (DSE)

DSE was measured using a modified version of the Maternal Self-Efficacy for Diabetes Management Scale (MSED) [28]. This scale was developed to measure the level of confidence with which parents or primary caregivers can independently manage diabetes-related performance tasks [7]. The scale includes 11 items rated on a 5-point Likert scale. There are three subscales, including diabetes management (2 items), problem solving (6 items), and teaching (3 items). A score of 1 indicates having no confidence all, and a score of 5 indicates being confident without help from others. The Cronbach’s alpha was 0.83 in the study by Noser et al. (2019) and 0.94 in the present study.

#### 2.2.5. Quality of Life (QOL)

QOL was measured using the Quality of Life in a Child’s Chronic Disease Questionnaire (QLCCDQ) [29]. This scale was developed to measure parental QOL in children aged 5–14 years with chronic diseases such as T1D or asthma. The scale comprises 15 items rated on a 7-point Likert scale. There are three subscales, including role functioning (9 items), emotions (3 items), and symptoms (3 items). A score of 1 indicates being very disturbed or restricted, and a score of 7 indicates not being restricted at all. The Cronbach’s alpha scores for the subscales were 0.77 to 0.93 in Farnik et al.’s (2010) study and was 0.94 in the present study.

#### 2.2.6. Demographic Measures

The demographic variables were developed based on prior literature that included a survey study of parents of children with T1D [9,12,30].

### 2.3. Ethical Considerations

This study was approved by the institutional review board (IRB) (1041386-202006-HR-32-02, 1041386-202009-HR-56-01). Participants voluntarily agreed to participate in the survey. The researcher posted the requirements, aims, and methods of this study in an online self-help group community and obtained informed consent from participants. 

### 2.4. Procedure

Following IRB approval, the author requested permission from the steering committee of “Sugartree”, a major online health community in South Korea, to host the study. Sugartree is a self-help group that includes approximately 2000 families of children with T1D. After obtaining permission, the author distributed a URL linked to the web survey via the online community’s virtual noticeboard, notes, and email. The self-rated questionnaire was designed using SurveyMonkey. Data were collected from 1 August to 24 September 2020. A coffee coupon was offered as a reward. Data were automatically coded in SurveyMonkey and exported to Excel files.

### 2.5. Data Analysis

Data were analyzed using IBM SPSS Statistics for Windows, version 25.0. Descriptive statistics were used. Univariate analysis using an independent *t*-test, one-way analysis of variance (ANOVA), and Pearson’s correlation were conducted to determine whether predictors should be included in the multiple regression analysis. Differences between QOL and demographic factors, excluding age and duration of illness, were analyzed using an independent *t*-test and ANOVA. A Pearson’s correlation was conducted to investigate the correlation between QOL, OHCE, OHSS, DSE, DRB, mother’s age, and the duration of the child’s illness. Multiple regression analysis was conducted to determine predictors of QOL using significant variables in univariate analysis. Variance inflation factor (VIF) and tolerance were checked to verify multicollinearity between predictors and QOL.

## 3. Results

### 3.1. Demographics of Participants 

A total of 208 questionnaires were completed. Demographic characteristics and child disease-related characteristics are presented in Table 1. The mean age of the participants was 42.1 years (SD = 4.3). 

Among the participants, 106 were employed, and most (70.2%) had university diplomas. Most participants (96.6%) were married. The majority of participants did not follow any religion (54.3%). Furthermore, 77.9% of participants had two children. The majority (48.6%) reported a family monthly income of over 5.5 million won (1000 Korean won is about 1 US dollar).

Most of the children’s diabetes management (91.3%) were conducted by their mothers. The mean duration of diabetes diagnosis in the children was 57.6 months (SD = 39.5). A total of 8.2% of children had other chronic comorbid conditions. The mean age of the children was 11.4 years (SD = 3.2). All except nine of the children were using continuous glucose monitoring (CGM). However, only 50.0% were using an insulin pump.

### 3.2. Mean Scores for OHCE, OHSS, DSE, DRB, and QOL

The mean score for the participants’ OHCE was 3.92 (SD = 0.47). The mean score for OHSS was 5.26 (SD = 1.13). The mean diabetes self-efficacy score was 3.59 (SD = 0.79). The mean QOL score was 4.72 (SD = 1.08) and the mean DRB score was 3.17 (SD = 0.73).

### 3.3. Differences in QOL According to Demographic Characteristics

Differences in QOL according to dichotomous or three nominal variables were analyzed using an independent *t*-test and ANOVA (see Table 2). The mean QOL of participants with jobs was significantly higher than those without jobs (*t* = 2.787; *p* = 0.006). The mean QOL was significantly lower for married mothers than divorced or widowed mothers (*t* = −2.319; *p* = 0.021). There were no differences observed in QOL regarding the other demographic characteristics.

Pearson’s correlation was used to examine the relationship between QOL and continuous demographic variables, namely OHCE, OHSS, DSE, and DRB (see Table 3). Assumptions for Pearson’s correlation were met. The results showed that the mean age of the children, OHCE, and DSE positively correlated with QOL (*r* = 0.192; *p* = 0.006, *r* = 0.201; *p* = 0.004, and *r* = 0.300; *p* < 0.001, respectively). DRB was negatively correlated with QOL *(r* = −0.794; *p* < 0.001). No significant relationships were found among the other variables.

### 3.4. Predictors of QOL

Employment status, caregiver status, the child’s grade, OHCE, DSE, and DRB were subjected to stepwise multiple linear regression analysis. The regression model’s basic assumptions, including linearity, no multicollinearity, multivariate normality, independence of the residuals (no autocorrelations), and homoscedasticity, were examined. First, scatterplots and correlations were checked to test linearity. To test multicollinearity, the variance inflation factor (VIF) was lower than 10 and tolerance was higher than 0.1, with a condition index score of less than 30. Second, to test normality, dots following the diagonal line in the P–P plot were examined. The *p*-value was not significant in the Kolmogorov–Smirnov test for the standardized residual (*p* = 0.20). Third, to test independence of errors, the dots scattered along the residual against QOL were checked. The Durbin–Watson value was 2.011. Finally, to test the normal distribution (homoscedasticity), the dots scattered along the residual against QOL were checked.

The findings showed that DRB (*β* = −0.783; *p* < 0.001) and mean age of children (*β* = 0.100; *p* < 0.019) can affect the QOL of mothers of children with T1D. This model explained 64% of the total variance of QOL of mothers using the online health community for children with T1D (*F* = 182.868; *p* < 0.001). DRB was the most powerful factor contributing to maternal QOL (see Table 4).

## 4. Discussion

Parental or caregiver QOL is a crucial factor in health outcomes for children and those with chronic diseases and complex illnesses. The present study employed a scale developed to measure the disease specific QOL of parents caring for children with chronic diseases such as T1D. This study found that the older age of children and lower DRB were predictors of higher QOL. This is consistent with previous research indicating that parents of young children were more worried than parents of children in older age groups [11]. In the present study, the mean age of participants’ children was 11.4 years, and their ages ranged from 6 to 18 years. A significant relationship was observed between the children’s ages and the parental QOL score [31]. This may be because younger children require parental assistance during insulin injection and insulin dose determination. In this study, 95.7% of the children used CGM, but 50.0% did not use an insulin pump. Accordingly, several insulin injections were required daily, although CGM provides convenience by reducing the need for blood sugar testing using a lancet. In particular, school-aged children spend many hours at school, and parents need to be involved in their healthcare during the school day. A previous study including students aged 6 to 9 years (grades 1 to 3) reported that parents frequently received calls from the school or were requested to attend school, and some parents directly administered their children’s insulin at school [32]. In another study, 82% of children aged 6 to 9 years were able to interpret blood sugar readings, but only 60% were able to inject insulin, and only 40% were able to determine the dosage and type of insulin [33]. Furthermore, while 56.5% of children aged 6 to 9 years required assistance in administering insulin injections, 94.7% of children aged 10 to 12 years and 97.4% of children aged 13 to 15 years administered their insulin injections independently at school [33]. Children being away at school or at a daycare facility, and subsequent concerns about hypoglycemia, were significantly associated with parental QOL [5]. For children in the lower grades, insulin injections are administered at least once before lunch time. Therefore, their mothers must visit school at least once a day, and these routine school visits have a significant impact on the mothers’ lives. The QOL of mothers of children in higher grades who can independently perform self-care is considerably better.

DRB was an independent predictor of parental QOL, which is supported by previous research [12]. This is similar to a finding that a higher level of caregiver burden was negatively associated with parental QOL in parents of children with asthma [34,35]. This finding is consistent with the QOL of mothers of children with chronic conditions being affected by emotional support, care dependency, vacation days, and parental chronic illness [13]. Interestingly, the total DRB score in this study was 57.1, which was higher than the score of 10 obtained in Markiwitz et al.’s study (2012). Since the mean age of children was 13 years in Markiwitz et al.’s (2012) study, they could have independently performed self-care. As a result, the DRB score would have been low. Notably, the correlation between QOL and DRB was in the range of −0.37 to −0.48 in Markowitz et al.’s study (2012), but the correlation in this study was −0.794, indicating a high correlation coefficient. This finding may also be attributed to the children in the present study being far younger than the children in Markowitz et al.’s study (2012). Three domains of QOL, role function, symptoms, and emotions, were correlated with immediate burden (*r* = −0.726, −0.567, and −0.649, respectively) and theoretical burden (*r* = −0.656, −0.619, and −0.555, respectively) in the present study. The correlation between the role function domain of QOL and immediate burden was the highest at −0.726. This may include mothers caring for children younger than those in a previous study, thus, the immediate increase in role burden and the decrease in QOL may have a greater correlation. Mothers’ DRB was shown to be significantly reduced after participation in a short parental group intervention, which included relaxation and guided visualization techniques [36]. The burden of care and anxiety about the future can also negatively affect mother’s QOL domains, including the domains of role function, emotions, and children’s symptoms. Farnik et al.’s study (2010) showed that mothers’ QOL was lower than that of fathers’ QOL in children with chronic diseases [29]. The QOL scale included the domains of role function, emotions, and symptoms. In Farnik et al.’s study (2010), mothers of children with chronic conditions including diabetes, asthma, and eczema reported role function of 4.65, emotions of 4.76, and symptoms of 2.85 in QOL scores [29]. However, in the present study, participants reported role function of 4.97, emotions of 4.28, and symptoms of 4.55. The symptoms score was higher than in Farnik et al.’s study (2010), while other domain scores were similar.

In the present study, primary caregivers were mothers of children with T1D, and 95.7% of children used CGM. This technology can detect early complications, such as hypoglycemia or hyperglycemia. Accordingly, the QOL score in the symptom domain may appear higher than that of mothers of children with other chronic diseases, such as asthma and eczema. Poorly controlled asthma symptoms negatively affected parental QOL [37]. Since this study did not investigate QOL for fathers, it is necessary to compare QOL of fathers of children with T1D. It is essential to provide skills and knowledge related to caring for children in lower grades, and to provide interventions for care-related difficulties.

Additionally, the QOL score was higher when the mother was employed and was the primary caregiver of the child with T1D in the present study. The higher QOL among employed mothers can be attributed to economic improvement and self-development through work. Previous research showed no significant differences in work–family conflict and work–family facilitation between mothers of children with T1D and controls [38]. In the literature, when the grandmother was the primary caregiver, the QOL score was lower than that of mothers of children with other chronic conditions [39]. The QOL perceived by fathers was significantly higher than that of mothers [29]. Previous research shows that family income influenced family hardiness [40]. However, there was no relationship between family income, primary caregiver, and maternal QOL in the present study.

There was no relationship between perceived OHSS and QOL in the present study, unlike the findings of a previous study [9]. Parents of children with T1D reduced uncertainty by joining a support group [41]. Social support for emotional, instrumental, and informational aspects showed a linear relationship with QOL in stressful situations; however, social support was not related to QOL in stress-free situations [42]. Previous research has shown that social support is directly related to caregiver QOL [43] or patient QOL [44]. In addition, OHSS scores of 5.26 in this study were higher than the score of 4.6 reported in another study by the online health community group using healthcare centers [26]. Parents of young children with special health requirements seek information to help cope with emotional challenges and seek social support from each other using social media [45]. Moreover, parents reported that parental support groups helped to address their concerns and enabled them to better recognize their children’s health needs. [46]. Further research is needed to elucidate the relationship between online social support and QOL in online health communities for families of children with T1D.

DSE was also positively correlated with maternal QOL but was excluded in the multivariate analysis. The total DSE score was 39.5 in the present study, which was lower than 44.7 reported in Noser et al.’s study (2019), which included parents of children with a mean age of 13.5 years. A previous study indicated that parental self-efficacy was associated with better parental QOL in young children with T1D, where the mean age was 6.6 years (*r* = 0.41, *p* = 0.002) [47]. However, the correlation coefficient was higher than the coefficient of 0.30 in the present study. Parental self-efficacy was correlated with emotional subcategories in health related QOL of parents of children with chronic illness and medical complications [48].

OHCE was also positively correlated with QOL but was excluded in the multivariate analysis. A strategy to enhance empowerment among patients with diabetes was related to an increase in diabetes-related knowledge, behaviors, and improvements in self-care [49]. In addition, empowerment-based interventions were associated with decreased distress related to emotions, medications, and health care professionals, and an increase in QOL [50]. Currently, online self-help groups for children with T1D are endeavoring to expand insurance coverage for children’s healthcare and to improve healthcare systems, and are advocating for children’s rights and interests [51]. Research on the relationship between OHCE and diabetes-related health outcomes for parents of children with T1D is currently lacking. In the current study, DSE and OHCE were excluded from multiple regression analysis with a strong effect of diabetic burden on QOL. This can provide evidence for intervention strategies to reduce the burden of diabetes management, while improving collective empowerment and DSE through online platforms.

The present study has some limitations. Family income, education level, CGM/insulin pump use rate, and type of CGM/insulin pump of all children with T1D in Korea are unknown. There is a possibility of non-coverage bias.

## 5. Conclusions

The current study demonstrated that mothers of children with T1D, who were enrolled in grades 1 to 12, felt a great burden in the daily management of diabetes. In the present study, it was found that DRB and the age of participants’ children were predictors of QOL in mothers of children with T1D. In mothers of younger children, it is important to reduce DRB. Factors related to DRB should be identified, and strategies to reduce DRB should be established. The findings indicated that OHCE and DSE are correlated with mothers’ QOL. It is also necessary to include strategies to promote OHCE and DSE as interventions to improve QOL of mothers of children with T1D.

## Figures and Tables

**Table 1:** Demographic characteristics (*N* = 208).

Variables		*n* (%) or M ± SD
Mother-related factors		
Age (years)		42.06 ± 4.30
Employment	Employed	106 (51.0)
	Not employed	102 (49.0)
Education	High school	39 (18.8)
	University	146 (70.2)
	Graduate school	23 (11.1)
Marital status	Married	201 (96.6)
	Divorced or widowed	7 (3.4)
Religion	No	113 (54.3)
	Yes	95 (45.7)
Number of children	≥2	162 (77.9)
	1	46 (22.1)
Family monthly income (in millions of Korean won)	≥5.5	101 (48.6)
	3 to 5.5	86 (41.3)
	<3	21 (10.1)
Primary caregiver	Mother	190 (91.3)
	Father or grandparents	18 (8.7)
Child-related factors		
Sex	Male	87 (46.6)
	Female	111 (53.4)
Age (years)		11.42 ± 3.22
Diabetes duration (months)		57.57 ± 39.53
Other chronic disease	No	191 (91.8)
	Yes	17 (8.2)
Continuous glucose monitoring	No use	9 (4.3)
	Use	199 (95.7)
Insulin pump	No use	104 (50.0)
	Use	104 (50.0)

SD = standard deviation.

**Table 2:** Differences in quality of life (QOL) according to demographic characteristics (*N* = 208).

Variables		*n*	M ± SD	*t* or *F*	*p*
Mother-related factors					
Employment	Employed	106	4.92 ± 1.03	2.787	0.006
	Not employed	102	4.51 ± 1.09		
Education	High school	27	4.63 ± 1.28	0.189	0.828
	University	107	4.74 ± 1.00		
	Graduate school	18	4.74 ± 1.21		
Marital status	Married	201	4.69 ± 1.07	−2.319	0.021
	Divorced or widowed	7	5.64 ± 0.91		
Religion	No	113	4.79 ± 1.03	0.959	0.339
	Yes	68	4.64 ± 1.13		
Number of children	≥2	162	4.74 ± 1.04	0.370	0.712
	1	46	4.67 ± 1.20		
Family monthly income (in millions of Korean won)	≥5.5	101	4.78 ± 0.96	0.935	0.394
	3 to 5.5	86	4.73 ± 1.24		
	<3	21	4.43 ± 0.83		
Primary caregiver	Mother	190	4.75 ± 1.07	1.083	0.280
	Father or grandparents	18	4.46 ± 1.16		
Child-related factors					
Sex	Male	97	4.75 ± 1.04	0.312	0.756
	Female	111	4.70 ± 1.11		
Other chronic disease	No	191	4.73 ± 1.06	0.423	0.673
	Yes	17	4.62 ± 1.31		
Continuous glucose monitoring	No use	9	5.07 ± 1.10	0.985	0.326
	Use	199	4.71 ± 1.07		
Insulin pump	No use	104	4.75 ± 1.04	0.399	0.690
	Use	104	4.69 ± 1.11		

**Table 3:** Correlation between continuous demographic variables, online health community collective empowerment (OHCE), online health community social support (OHSS), diabetes self-efficacy (DSE), and diabetes-related burden (DRB), and QOL (*N* = 208).

Variables	*r*	*p*
Maternal Age (years)	0.015	0.827
Child age (years)	0.192	0.006
Disease period (months)	0.113	0.103
Online health community collective empowerment	0.201	0.004
Online health community social support	0.060	0.388
Diabetes self-efficacy	0.300	<0.001
Diabetes-related burden	−0.794	<0.001

**Table 4:** Predictors of maternal QOL for children with type 1 diabetes (T1D) in an online health community (*N* = 208).

Variables	*b*	*β*	*t*	*p*	R^2^	Adjusted R^2^
Constant	7.975		29.389		0.641	0.637
Diabetes-related burden	−1.146	−0.783	−18.567	<0.001		
Child age	0.330	0.100	2.370	0.019		
